# CoVennTree: a new method for the comparative analysis of large datasets

**DOI:** 10.3389/fgene.2015.00043

**Published:** 2015-02-20

**Authors:** Steffen C. Lott, Björn Voß, Wolfgang R. Hess, Claudia Steglich

**Affiliations:** ^1^Genetics & Experimental Bioinformatics, Faculty of Biology, University of FreiburgFreiburg, Germany; ^2^Computational Transcriptomics, Faculty of Biology, University of FreiburgFreiburg, Germany

**Keywords:** CoVennTree, weighted Venn diagram, VDS value, massive comparative analysis, rooted tree

## Abstract

The visualization of massive datasets, such as those resulting from comparative metatranscriptome analyses or the analysis of microbial population structures using ribosomal RNA sequences, is a challenging task. We developed a new method called CoVennTree (Comparative weighted Venn Tree) that simultaneously compares up to three multifarious datasets by aggregating and propagating information from the bottom to the top level and produces a graphical output in Cytoscape. With the introduction of weighted Venn structures, the contents and relationships of various datasets can be correlated and simultaneously aggregated without losing information. We demonstrate the suitability of this approach using a dataset of 16S rDNA sequences obtained from microbial populations at three different depths of the Gulf of Aqaba in the Red Sea. CoVennTree has been integrated into the Galaxy ToolShed and can be directly downloaded and integrated into the user instance.

## 1. Introduction

In recent years, new high-throughput sequencing technologies such as 454, Illumina and SOLiD have become available and have led to an enormous increase in the volume of available sequence data while simultaneously facilitating a dramatic decrease in sequencing costs. The development of these technologies has enabled the large-scale application of metatranscriptomics and metagenomics approaches and has been responsible for substantial advances in a broad variety of research, including the large-scale identification of DNA polymorphisms, investigations of the compositions of microbial communities, and genome- and population-wide gene expression studies at single-nucleotide resolution. For the first time, the comprehensive comparison of sequences obtained in the field with sequences from databases using annotated functions has become possible and has enabled the assessment of environmentally important genes and their linked metabolic pathways. The first step in the analysis of sequencing data is based on either a composition or a comparison approach. The latter consists of the mapping of reads against a database using BLAST (Altschul et al., 1990), followed by an assignment algorithm that assigns the reads to their corresponding taxonomy groups. The result is a tree-like data structure that contains a specific number of reads for every group. The taxonomy tree is a rooted tree with nodes and edges that are well-ordered and allows for distinguishing between distinct groups, such as kingdoms and phyla, down to the species level. To date, the NCBI taxonomy tree contains more than 22,928 entries for “higher taxa” and over 444,254 entries for “total taxa” (January 28, 2015). A BLAST search against such a complex database is time-consuming; moreover, complex datasets are also difficult to visualize in a comparative way. Several groups have developed visualization tools that can analyze large datasets, such as MEGAN (Huson et al., 2007), Krona (Ondov et al., 2011), BLASTatlas (Hallin et al., 2008), and MetaSee (Song et al., 2012); however, all of these applications are subject to limitations in one aspect or another. For instance, the graphical presentation may suffer from a lack of information; alternatively, with the addition of more details, the graphs may become difficult to interpret and impossible to present on a single printed page. An uncollapsed tree down to the leaf level is usually bushy and deeply branching and contains information concerning the relationships (diversity and similarity among leaves) of every single leaf with every other. A possible approach to reducing the complexity of the presented data without losing important information, or even increasing in complexity, is to combine scalable weighted Venn diagrams with a tree structure in which every node is transformed into a weighted Venn diagram and the leaf information is condensed by grouping related child leaves at a higher level toward the root node. Additionally, the size of the weighted Venn circles can be correlated with the number of members that belong to a node, and up to three datasets can be compared in a single weighted Venn diagram. Here, we have developed a new method called CoVennTree (*Co*mparative weighted *Venn Tree*) that compares up to three datasets by aggregating and transferring information from the bottom to the top level and produces a graphical output in Cytoscape (Shannon et al., 2003). The underlying concept of CoVennTree is to bring information from the leaf level up to the root node while maintaining the properties of the content of every dataset. With the introduction of weighted Venn structures, the amounts and relationships of data associated with different conditions can be correlated and simultaneously aggregated without losing relevant information.

## 2. Methods

### 2.1. Definition of weighted Venn computation

A weighted Venn data structure for three datasets is completely defined by a 6-tuple (*w*_1_,*w*_2_,*w*_3_,*w*_1,2_,*w*_1,3_,*w*_2,3_), where *w_i_* is the weight for condition *i* and *w_i,j_* is the weight of the co-occurrence of conditions *i* and *j*. To compute a parent weighted Venn diagram, all relevant children are summed. The initial leaf weights are the raw counts for the corresponding conditions. For values of *w*_1_ = 1000, *w*_2_ = 3000, and *w*_3_ = 4000, the co-occurrence weights are *w*_1,2_ = 1000, *w*_1,3_ = 1000, and *w*_2,3_ = 3000. The resulting weighted Venn diagram for each leaf contains three interleaving circles, which overlap by 100%.

### 2.2. Definition of the weighted Venn decomposition similarity (VDS) value

Prior to the VDS calculation, three sets are defined as follows: “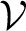 := the set of weighted Venn diagrams (children) for a corresponding parent,” “
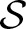
(*x*) := the number of conditions with a weight greater than zero for any child of node *x*” and “
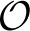
(*x*) := the number of conditions with co-occurrence weights greater than zero for any child of node *x*.”

To compute the VDS value for the given children, five steps are required (Equation 1). The two sums in Equation (1) represent the decomposition of the weighted Venn diagrams: the first sum is related to the total content of every dataset, and the second sum is related to the overlaps between different datasets. The maximum number of datasets or possible overlaps is three; therefore, the sums run from 1 to 3. To normalize the values to an interval of [0, 1], the outcome of each sum is divided by its corresponding set, |
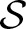
| or |
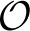
|. Summing both values then increases the relevant interval from [0, 1] to [0, 2], necessitating multiplication by $\frac{1}{2}$ to transform the value back to the interval [0, 1]. The result is assigned to the corresponding parent node and characterizes the similarity among the children in size and structure.

Equations (2) through (5) describe the essential steps that are involved in the decomposition in detail. In this context, decomposition means the splitting of every child node (weighted Venn diagram) into two vectors. One vector contains the number of data points in every dataset (called weights), and the other contains the numbers of data points that are shared between datasets 1 and 2, between datasets 1 and 3, and between datasets 2 and 3 (called co-occurrence weights). All vectors of the children of a parent node are stored in a corresponding matrix. Matrix Θ contains all sets, and matrix Π contains all overlaps. Every column ϑ_1*n*_, ϑ_2*n*_, and ϑ_3*n*_ in matrix Θ is related to a corresponding column in matrix Π: π_1*n*_, π_2*n*_, and π_3*n*_, respectively. Every row in matrix Θ corresponds to a condition, and every row in matrix Π corresponds to a co-occurrence (the co-occurrence of conditions 1 and 2, the co-occurrence of conditions 1 and 3 or the co-occurrence of conditions 2 and 3). The information contents of the matrices Θ - Π, Θ′ - Π′, Θ″ - Π‴, and Θ‴ - Π‴ are distinct, but the mathematical operations are the same for each step.

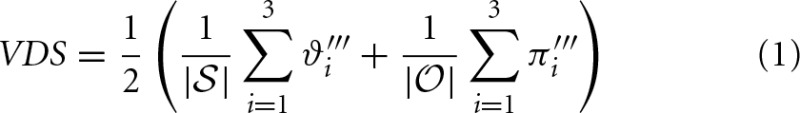



In Equation (2), the variables ϑ_*i*._ and π_*i*._ for *i* ϵ 1, 2, 3 contain the sum of every row. These quantities are used to compute a ratio for every entry in matrices Θ′ and Π′, and these ratios reveal the degrees of correlation between specific datasets. Thereafter, every row is summed, and the outcomes are stored to the variables ϑ′_*i*._ and π′_*i*._ for *i* ϵ 1, 2, 3 (see Equation 3). Equation (4) represents a condensation step and reduces the matrix dimension from 3× *n* to 3 × 1 (where *n*: = number of children) using the outcome of the previous step. Finally, a normalization step is required to bring the values into the interval [0, 1] (see Equation 5). Then, the values ϑ‴_*i*_ and π‴_*i*_ for *i* ϵ 1, 2, 3 can be used to compute the final value (Equation 1).

(2) $$\Theta =\left[\begin{array}{cccc} \vartheta_{11} & \vartheta_{12} & \cdots & \vartheta_{1n}\\ \vartheta_{21} & \vartheta_{22} & \cdots & \vartheta_{2n}\\ \vartheta_{31} & \vartheta_{32} & \cdots & \vartheta_{3n} \end{array}|\begin{array}{c} \vartheta_{1.}\\ \vartheta_{2.}\\ \vartheta_{3.} \end{array}\right]\;\Pi =\left[\begin{array}{cccc} \pi_{11} & \pi_{12} & \cdots & \pi_{1n}\\ \pi_{21} & \pi_{22} & \cdots & \pi_{2n}\\ \pi_{31} & \pi_{32} & \cdots & \pi_{3n} \end{array}|\begin{array}{c} \pi_{1.}\\ \pi_{2.}\\ \pi_{3.} \end{array}\right]$$

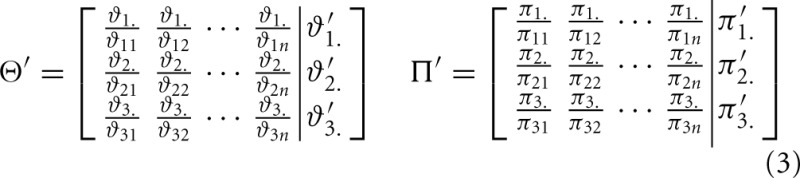

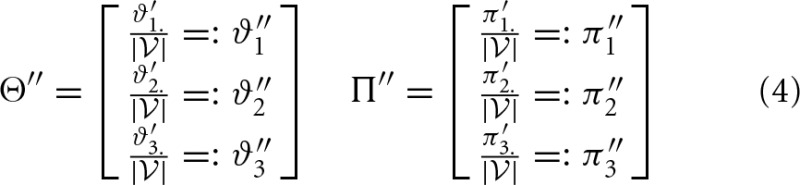

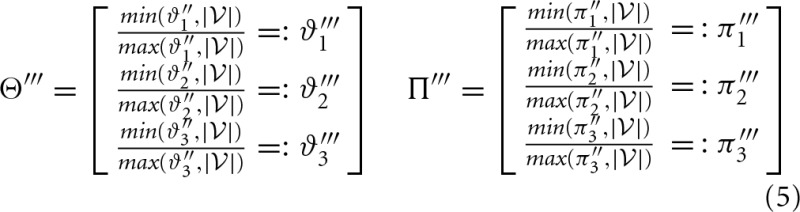

### 2.3. Description of frame computation

The following formulas (Equations 6–11) represent the procedure used to compute the frame size (space), which is essential for drawing a weighted Venn diagram. The graphical output, consisting of a weighted Venn diagram, is achieved by applying the Google API, but this tool does not allow for the manual adjustment of the position of a single set. Therefore, a combination of the complete sums [*f*(node_sum_)] and the overlaps with the largest set [*f*(add_sum_)] is required to determine the frame size in pixels (Equation 6). The function *f*(*x*) allows for the transformation of a large number range into an integer value and thus renders visualization feasible. To determine the value of node_sum_, the available sets for the corresponding weighted Venn diagram are summed (Equation 8).

For instance, if only the first two sets are available, the final set (3 of 3) takes a value of zero and does not contribute to the outcome. The additional value add_sum_ represents the region in which there is no overlap between the largest set and the remaining smaller sets, which is incorporated into the weighted Venn diagram structure. Equation (9) returns the sum of the smaller sets, and Equation 10 returns the overlap between the largest set and the smaller sets. The non-overlapping component is determined by subtracting corr_ov_ from corr_set_, and this additional value add_sum_ is used to expand the native frame size.

(6) $$\text{frame}=f({\text{node}}_{\text{sum}})+f({\text{add}}_{\text{sum}})$$

(7) $$f(x)=\{\begin{array}{ll} \left\lfloor 1.8\sqrt[{1.6}]{x}\right\rfloor +8 & \text{if}\sum_{i\;=1}^{3}w_{i}\leq 3,000\\ \left\lfloor 1.8\sqrt[{2.1}]{x}\right\rfloor +8 & \text{if}\sum_{i\;=1}^{3}w_{i}\leq 30,000\\ \left\lfloor 1.8\sqrt[{2.6}]{x}\right\rfloor +8 & \text{if}\sum_{i\;=1}^{3}w_{i}\leq 300,000\\ \left\lfloor 1.8\sqrt[{3.1}]{x}\right\rfloor +8 & \text{if}\sum_{i\;=1}^{3}w_{i}\leq 3,000,000\\ \left\lfloor 1.8\sqrt[{3.7}]{x}\right\rfloor +8 & \text{if}\sum_{i\;=1}^{3}w_{i}\leq 30,000,000\\ \left\lfloor 1.8\sqrt[{4.0}]{x}\right\rfloor +8 & \text{if}\sum_{i\;=1}^{3}w_{i}\leq 300,000,000\\ \left\lfloor 1.8\sqrt[{4.7}]{x}\right\rfloor +8 & \text{if}\sum_{i\;=1}^{3}w_{i}\leq 3,000,000,000 \end{array}$$

(8) $${\text{node}}_{\text{sum}}=\sum_{i=1}^{3}w_{i}$$

(9) $${\text{corr}}_{w}=\{\begin{array}{ll} w_{2}+w_{3} & \text{if }w_{1}=\underset{i\in \{1,2,3\}}{\max}(w_{i})\\ w_{1}+w_{3} & \text{if }w_{2}=\underset{i\in \{1,2,3\}}{\max}(w_{i})\\ w_{1}+w_{2} & \text{if }w_{3}=\underset{i\in \{1,2,3\}}{\max}(w_{i}) \end{array}$$

(10) $${\text{corr}}_{ov}=\{\begin{array}{ll} w_{1,2}+w_{1,3} & \text{if }w_{1}=\underset{i\in \{1,2,3\}}{\max}(w_{i})\\ w_{1,2}+w_{2,3} & \text{if }w_{2}=\underset{i\in \{1,2,3\}}{\max}(w_{i})\\ w_{1,3}+w_{2,3} & \text{if }w_{3}=\underset{i\in \{1,2,3\}}{\max}(w_{i}) \end{array}$$

(11) $${\text{add}}_{\text{sum}}={\text{corr}}_{w}-{\text{corr}}_{\text{ov}}$$

## 3. Results

### 3.1. Principles and workflow

**Figure 1:**
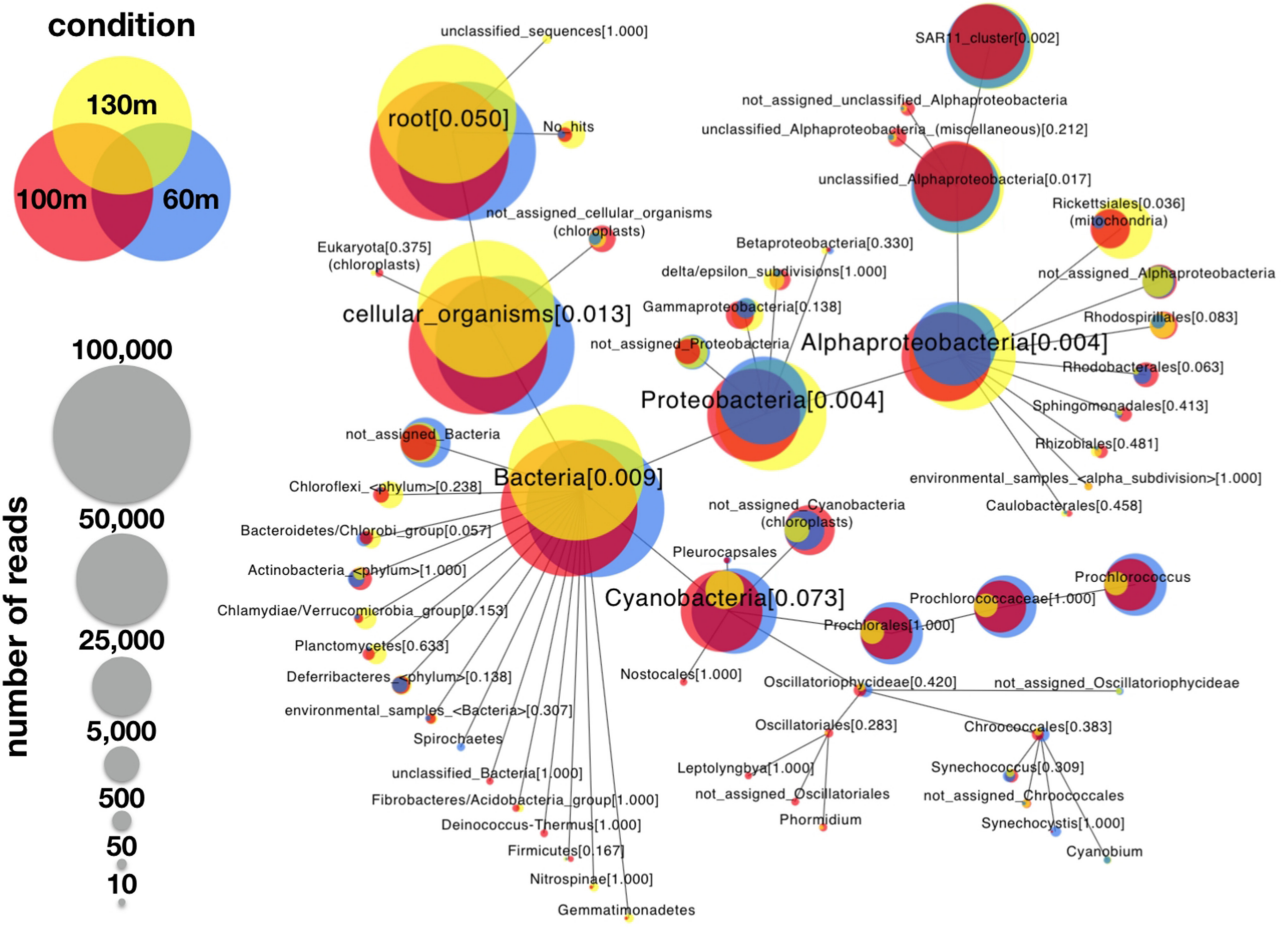
**Comparative weighted Venn tree based on partial 16S rRNA gene sequences of seawater samples from the Red Sea at 60 m (blue circles), 100 m (red circles), and 130 m (yellow circles)**. The tree was computed using CoVennTree. The numbers in parentheses refer to VDS values. The overlap of weighted Venn circles of parental nodes reflects sequence reads originating from the same organism (group). The libraries were normalized to 100,000 reads, and singletons were excluded from the analysis.

**Figure 2:**
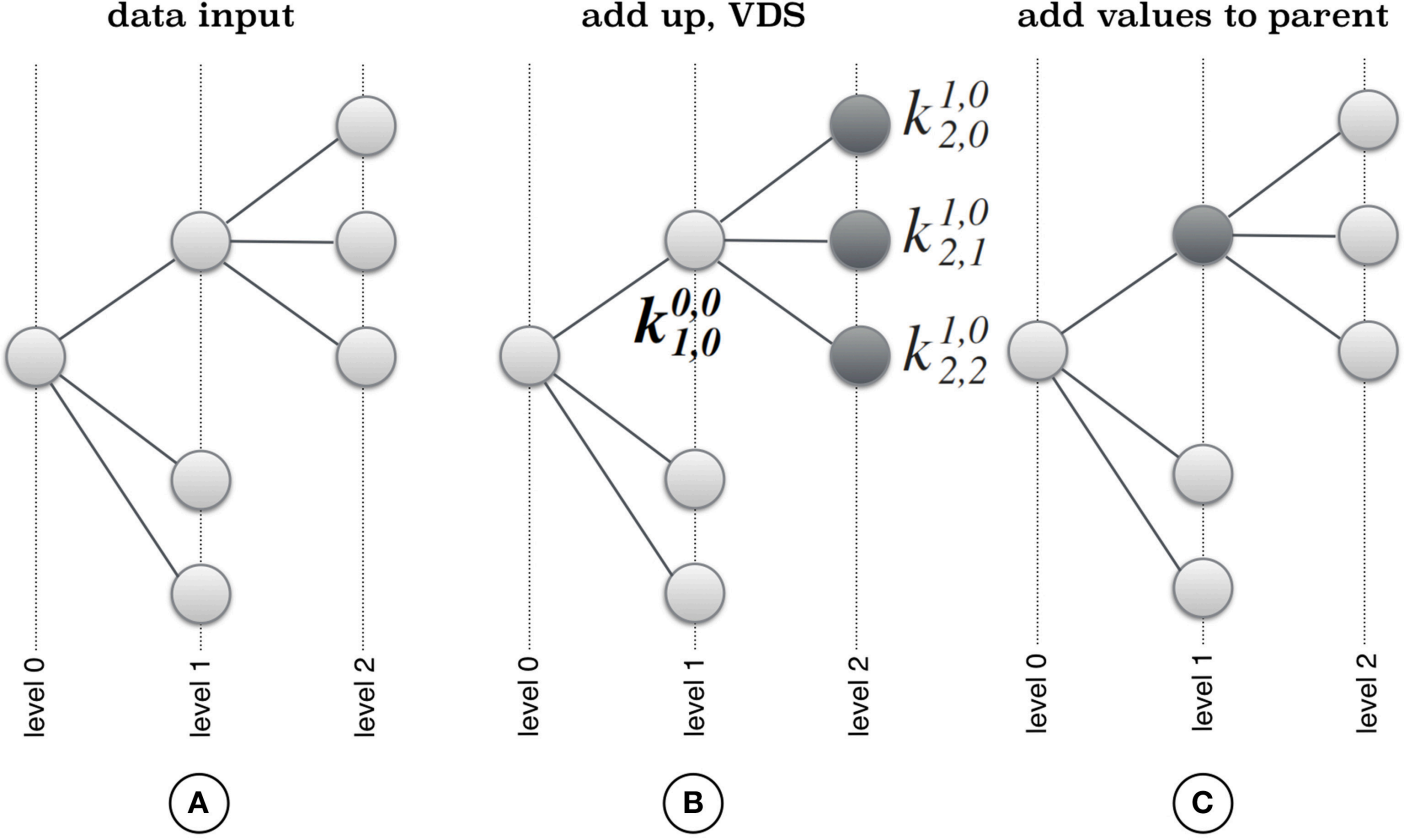
**Principle of CoVennTree. (A)** The algorithm starts with transforming the input path file into a rooted tree structure and computes weighted Venn diagrams for all leaves. **(B)** In a second step the parent weighted Venn diagram is computed by summing up all leaf weighted Venn diagrams. **(C)** In a final step all computed values for leaf diagrams are added up to the parent node until the root node is reached and the algorithm terminates.

**Figure 3:**
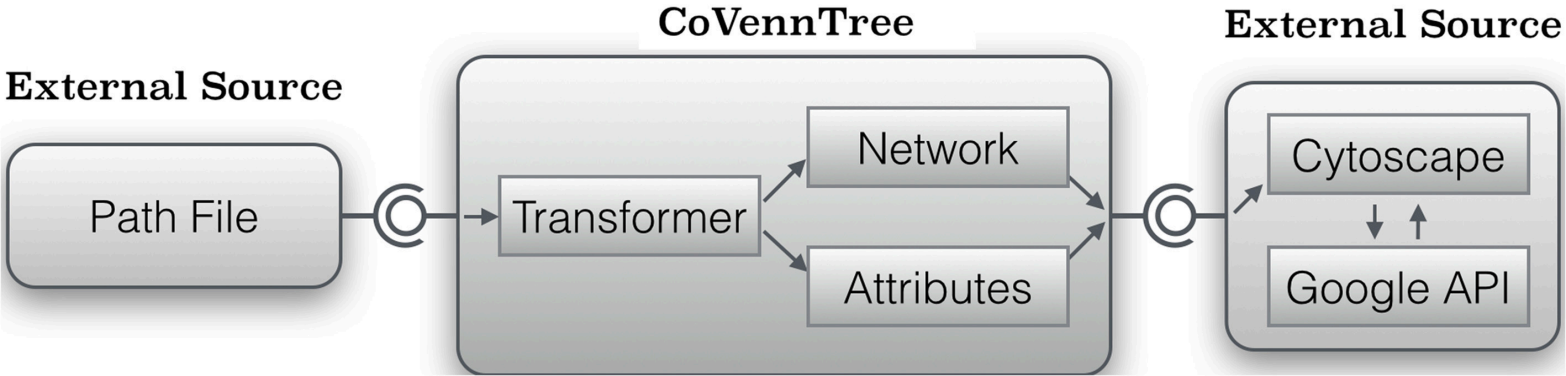
**Abstract model to create a weighted Venn tree from a given path file**. CoVennTree requires an input path file for the transformation tool CoVennTree, which produces two new files. One file includes the entire network of the tree (network.sif) and the second one contains the attributes (attribute.venn) to describe every node. Cytoscape processes both files and creates a weighted Venn graph by using the URL attribute.

CoVennTree associates rooted tree data structures with weighted Venn diagrams to produce an aggregated and comparative tree visualization for up to three massive datasets (Figure 1; for more details, see section below). The first step of computation requires a rooted tree as input; this tree can be calculated using either MEGAN (Huson et al., 2007) or other sources (Figure 2). The calculation begins at the deepest level (here, level 2; see Figure 2A) by summing all children to their corresponding parent nodes (Figure 2B) and simultaneously calculating the weighted Venn decomposition similarity (VDS) value for every parent (Equation 1, Figure 2C). The VDS value expresses the similarity among the children in terms of datasets used, co-occurrences and weights. If these properties are identical for all children associated with a given parent, then the VDS value is 1. The previous steps are repeated until the algorithm reaches the root node and terminates. The workflow depicted in Figure 3 describes the steps required for the final visualization of CoVennTree. For the calculation of the tree, an external path file is used to create a network file and the associated attribute file. The input file contains a header line with the corresponding path and value information (for an example, see the Supplementary Material, Figure S1). The tree can be visualized in Cytoscape v2.8.x (Shannon et al., 2003), which uses both files and communicates directly with the Google application programming interface (API) to create the weighted Venn diagrams for every node in the tree. In the near future we will be presenting a new visualization plug-in that allows to perform the entire workflow in Galaxy. CoVennTree has been integrated in the Galaxy ToolShed (Blankenberg et al., 2014) and can be directly downloaded and integrated into the user's Galaxy instance (Giardine et al., 2005; Blankenberg et al., 2010; Goecks et al., 2010). Step-by-step video tutorials for the generation of CoVennTree graphs are available in the Supplementary Material, Files S1–S3.

### 3.2. VDS value

We developed a new correlation measure named the VDS (*V*enn *d*ecomposition *s*imilarity) value. The VDS value is computed based on child information and specifies how similar the children are in structure (position of the circles), size (number of data points per dataset) and data content with respect to their parents. If the VDS value is 1, then the structure of the children are identical to the parent. One of the key characteristics of CoVennTree is that a few nodes from the root level contain the complete information of all subsequent nodes. When one is working with large datasets, which produce complex tree structures, only a subset of the entire tree can be visualized in detail. However, the VDS value evaluates the similarity between a parent weighted Venn structure and its children, thereby enabling the estimation of the weighted Venn structures of the hidden child layer. The benefits of the VDS value become obvious in the analysis of large datasets. Our weighted Venn tree, with 277 nodes, is relatively small compared with the typical volumes of metatranscriptome data, which can constitute up to tens of thousands of nodes.

### 3.3. Application and comparison with established methods

**Figure 4:**
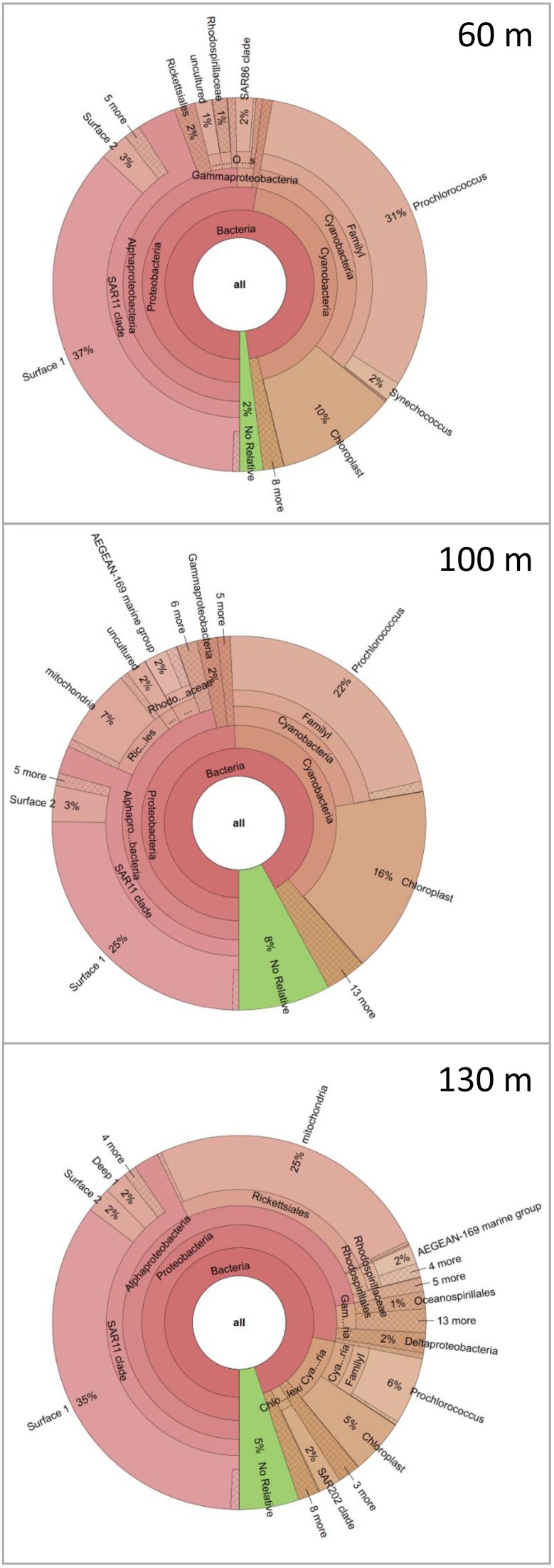
**16S rDNA sequence analysis of the three water samples using the SILVA database**. The taxonomic distributions of the marine communities are visualized in three individual Krona charts for 60 m, 100 m, and 130 m.

**Figure 5:**
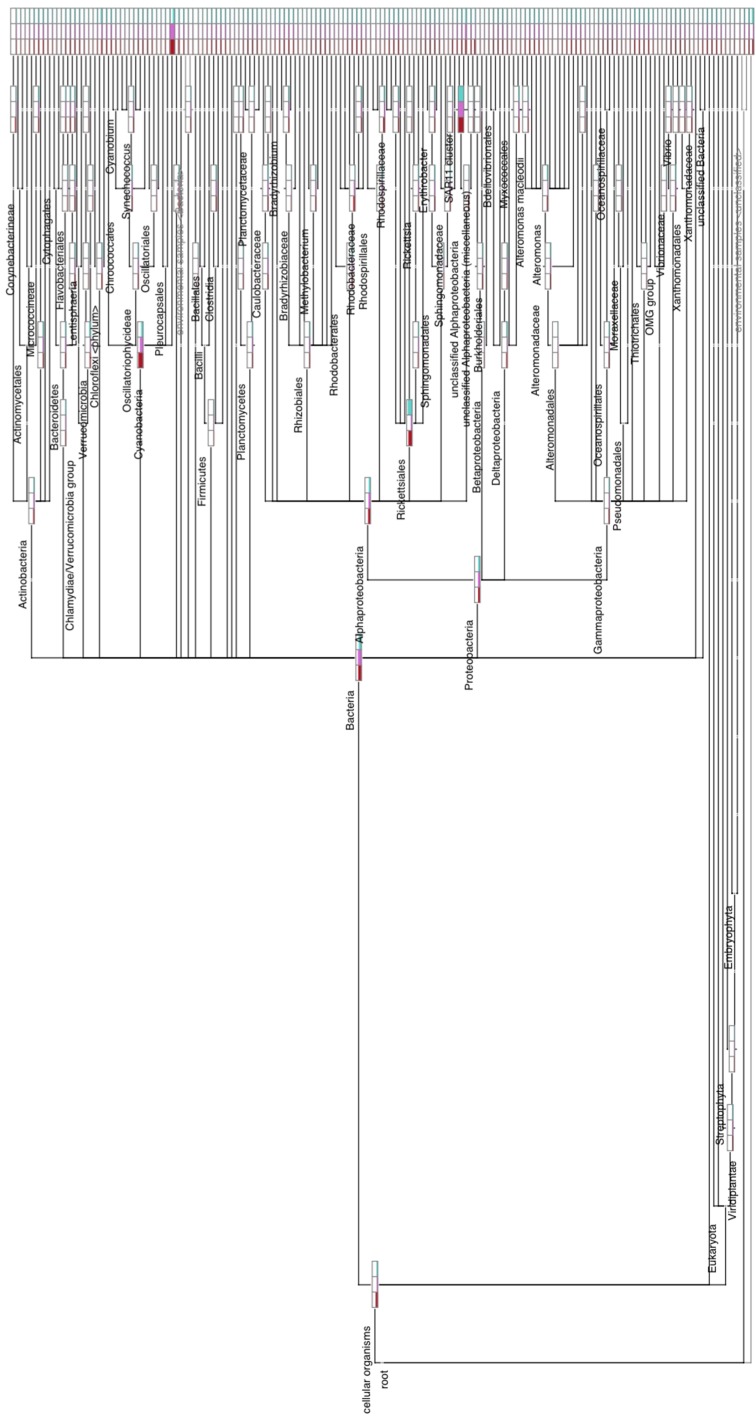
**Complete, uncollapsed rooted tree constructed by MEGAN version 5**. This graph contains all three datasets, represented by the bars for each node, and illustrates the number of reads assigned. Note that the different library sizes of the three datasets were not normalized to produce this figure.

To demonstrate the power of CoVennTree and illustrate its use, a comparative analysis was performed using three 16S rDNA datasets containing more than 150,000 sequences. Sampling for the 16S rDNA analysis was performed at station A in the Red Sea at depths of 60 m, 100 m, and 130 m. The processing of the samples has been described by Steglich et al. (2014). For phylogenetic classification, all sequence reads were compared against the SILVA database using BLASTn with the following settings: *E*-value 1e-5, dc-megablast. The BLAST results were further processed following the workflow described above or using the SILVA database (http://www.arb-silva.de/). For better comparison, each dataset was normalized to 100,000 reads. The graphical output of a CoVennTree result produced from these data is presented in Figure 1. CoVennTree assigns a specific color to each dataset and offers a choice among five color schemes (see the CoVennTree application in Galaxy; here, the 60 m data are shown in blue, the 100 m data in red, and the 130 m in yellow). For better comparison with the results from SILVA, only 49 of the 277 nodes that were determined by MEGAN are shown in Figure 1. Every node possesses a taxonomy label, and every parent node also lists the VDS value. Terminal nodes, which typically correspond to the species level, do not possess a VDS value because the calculation of the VDS value begins with these nodes and proceeds toward the root level. For example, the species *Prochlorococcus* belongs to the family Prochlorococcaceae, the order Prochlorales, and the phylum Cyanobacteria. Because Cyanobacteria other than *Prochlorococcus* were present at the sampling site and their depth distributions differed considerably from that of *Prochlorococcus*, the VDS value for Cyanobacteria is very low (VDS = 0.082). In contrast, the VDS values for Prochlorales and Prochlorococcaceae are each equal to 1.0, the maximal value, implying that all members of the order leaf and the family leaf belong to the species *Prochlorococcus*. This result explains why the weighted Venn diagrams from the order Prochlorales down to the species level are identical. The depth distribution of *Prochlorococcus* is comparable to the enumeration of the same samples via flow cytometry (Steglich et al., 2014). The highest cell numbers and the majority of 16S rDNA reads of *Prochlorococcus* were observed at 60 m. However, the majority of sequence reads were mapped to the phylum Proteobacteria, of which the alphaproteobacterial clade SAR11 constituted the most numerically abundant group. These results are consistent with previous reports, which have consistently found SAR11 to be the numerically dominant group in the marine environment (Rapp and Giovannoni, 2003; Schattenhofer et al., 2009; Thompson et al., 2013). A database that is frequently used for the analysis and visualization of ribosomal sequences is SILVA. SILVA-processed data can be presented as Krona plots (Figure 4) or “taxonomic fingerprint” plots (data not shown). The database is excellently curated; however, it does not contain all of the ribosomal reads that have been deposited, for instance, at NCBI. Within each Krona plot, data from a single sample can be visualized. Although Krona provides an intuitive overview of the data from every individual sample, it does not provide direct information regarding the correlation between different datasets in terms of read numbers and sequence content within a node. Therefore, changes in composition between different samples are not easily captured. In contrast, MEGAN (Huson et al., 2007) allows more than one dataset to be compared in a single graph and visualizes each dataset as a single bar in a chart diagram. The relative number of reads for a specific taxon is represented through the height of the bar. Figure 5 visualizes the complete, uncollapsed rooted tree for the three conditions and exemplifies the various problems encountered when this type of visualization style is used. Although the graph contains only 277 nodes (note that a metatranscriptome analysis can easily produce more than 25,000 nodes), it is not suitable for visualization on a single printed page. The most obvious disadvantage of MEGAN compared with CoVennTree is that the datasets cannot be correlated. For small datasets, a manual inspection of every taxon may be possible; however, the interpretation of large volumes of data by eye is not very practicable and is very time-consuming if not impossible. CoVennTree is able to overcome these limitations and integrates all information into a single weighted Venn diagram instead of computing three separate graphs; it therefore serves as an excellent complement to the existing set of well-established visualization tools.

## 4. Discussion

Producing clear, publication-ready trees for large datasets that can be presented on a single printed page is not a simple task. Most attempts focus on the extensive analysis of single datasets (for example, Krona Ondov et al., 2011) or compare only relative numbers of members per node for several datasets (for example, MEGAN (Huson et al., 2007) but in a non-printable format. Most importantly, none of the existing attempts can correlate all data points among several datasets in a comparative fashion. CoVennTree addresses these limitations by introducing weighted Venn diagrams, which visualize the number and correlation of members per node for each dataset. The adoption of a new method for calculating the similarity among sets in a weighted Venn diagram (defined by the VDS value) enables the computation of the diversity/similarity among children. The determination of the VDS value allows for the estimation of relationships between parents and their corresponding children at all tree levels. Our approach can be used for all rooted tree data structures that include multiple different conditions. For example, the “UPGMA” algorithm (Sokal and Michener, 1958) could be used to create a phylogenetic tree that contains several conditions per node. In this case, each condition corresponds to a circle in a weighted Venn diagram. An obvious limitation of our new method is that it can be used to analyze a maximum of three datasets. A typical Venn diagram is drawn in congruent circles and information about data size and intersection is given by numbers. This “static” approach allows to use more than three circles (datasets) in one Venn diagram. However, CoVennTree was developed to offer a graphical representation of data size (size of the circle) and intersection (overlap between datasets), which cannot be arranged for more than three datasets.

## Author contributions

SCL, BV, and CS conceived the tool. SCL developed the tool. CS and WRH performed the experiments. SCL, BV, WRH, and CS wrote the paper.

### Conflict of interest statement

The authors declare that the research was conducted in the absence of any commercial or financial relationships that could be construed as a potential conflict of interest.

## Supplementary material

Figure S1 **Example formats for the CoVennTree input file and the two corresponding output files**. The input file contains a header line; for three samples, the header reads as follows: #Datasets set1 set2 set3. The input file also contains a separate path for every node: “root;A;C;” 600,000 300,000 500,000. All words or values in the header line and path line are tab-delimited. The file is used as the input file for CoVennTree. The network file (network.sif) includes the entire network of the tree, and the attribute file (attribute.venn) contains the attributes to describe every node.

File S1 **The movie “Part1-MEGAN5” shows the merging of the tree and the extraction of the tree structure together with the number of sequences**.

File S2 **The movie “Part2-Galaxy” describes the uploading of the data into a Galaxy instance and the application of CoVennTree to the imported MEGAN files. Before an analysis can be performed, CoVennTree must be installed in a running Galaxy instance**.

File S3 **The movie “Part3-Cytoscape2.8” illustrates the uploading of the network file (^*^.sif) and the corresponding attribute file (^*^.venn) into Cytoscape version 2.8.x and provides an example of graph structuring**.
