# Supplementary material for: CoVennTree: a new method for the comparative analysis of large datasets
Source: Front Genet. 2015 Feb 20;6:43. doi: 10.3389/fgene.2015.00043 (PMC4335276; doi:10.3389/fgene.2015.00043)

— Input for CoVennTree —

#Datasets	set1	set2	set3
“root;”	0	0	0
“root;A;”	10000	0	0
“root;A;C;”	600000	300000	500000
“root;A;D;”	0	100000	200000
“root;A;E;”	800000	0	100000
“root;B;”	10000	20000	50000

separated by 1 tab

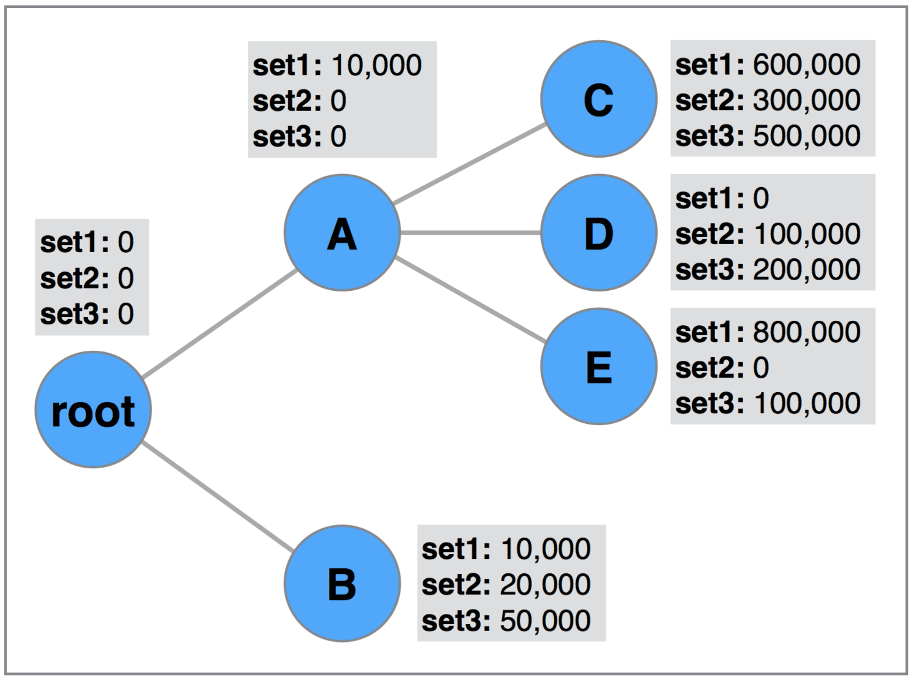

— Output CoVennTree Network —

```
root pp A
A    pp C
A    pp D
A    pp E
root pp B
```

— Output CoVennTree Attributes (Figure B) —

```
root   http://chart.apis.google.com/chart?chs=339x339&chco=FF2A00,00CCFF,9CFF00&cht=v&chd=t:
52.8,31.6,15.6,22.7,11.5,15.6,0.0&chf=bg,s,e0dede00root[0.134] 1420000 420000 850000 310000 610000 420000 0
A      http://chart.apis.google.com/chart?chs=332x332&chco=FF2A00,00CCFF,9CFF00&cht=v&chd=t:
54.0,30.7,15.3,23.0,11.5,15.3,0.0&chf=bg,s,e0dede00A[0.357]    1410000 400000 800000 300000 600000 400000 0
B      http://chart.apis.google.com/chart?chs=84x84&chco=00CCFF,9CFF00,FF2A00&cht=v&chd=t:
62.5,25.0,12.5,25.0,12.5,12.5,0.0&chf=bg,s,e0dede00     10000 20000 50000 10000 10000 20000 0
not_assigned_A       http://chart.apis.google.com/chart?chs=51x51&chco=FF2A00,9CFF00,00CCFF&cht=v&chd=t:
100.0,0.0,0.0,0.0,0.0,0.0,0.0&chf=bg,s,e0dede00  not_assigned_A       10000 0 0 0 0 0 0
C      http://chart.apis.google.com/chart?chs=188x188&chco=FF2A00,00CCFF,9CFF00&cht=v&chd=t:
42.9,35.7,21.4,35.7,21.4,21.4,0.0&chf=bg,s,e0dede00600000 300000 500000 300000 500000 300000 0
D      http://chart.apis.google.com/chart?chs=121x121&chco=00CCFF,9CFF00,FF2A00&cht=v&chd=t:
66.7,33.3,0.0,33.3,0.0,0.0,0.0&chf=bg,s,e0dede00 0 100000 200000 0 0 100000 0
E      http://chart.apis.google.com/chart?chs=165x165&chco=FF2A00,00CCFF,9CFF00&cht=v&chd=t:
88.9,11.1,0.0,11.1,0.0,0.0,0.0&chf=bg,s,e0dede00 800000 0 100000 0 100000 0 0
```

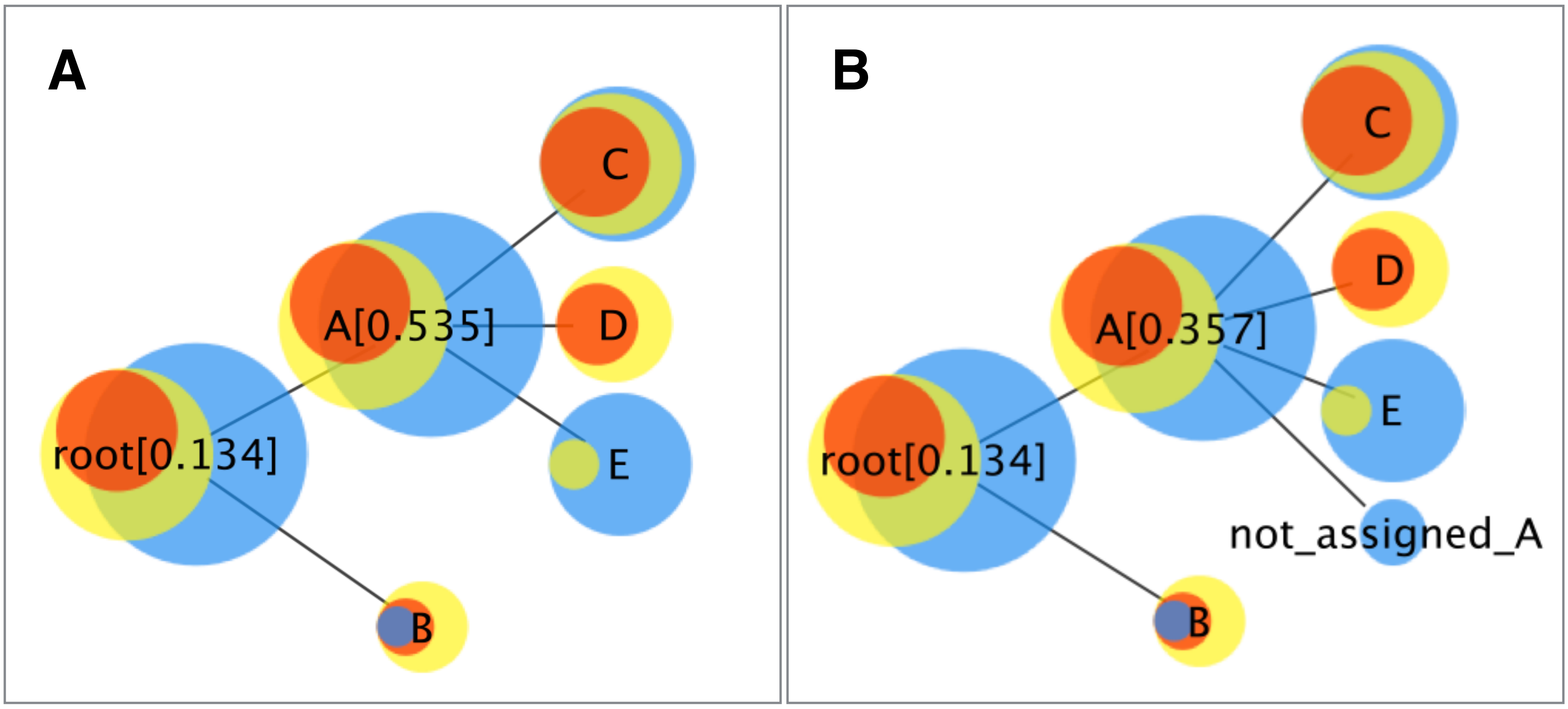

Supplement: Figure S1 — Example formats for the CoVennTree input file and the two corresponding output files. The input file contains a header line; for three samples, the header reads as follows: #Datasets set1 set2 set3. The input file also contains a separate path for every node: “root;A;C;” 600,000 300,000 500,000. All words or values in the header line and path line are tab-delimited. The file is used as the input file for CoVennTree. The network file (network.sif) includes the entire network of the tree, and the attribute file (attribute.venn) contains the attributes to describe every node. [file Image1.PDF]
